# Long-Term Mosquito culture with SkitoSnack, an artificial blood meal replacement

**DOI:** 10.1371/journal.pntd.0008591

**Published:** 2020-09-17

**Authors:** Yashoda Kandel, Soumi Mitra, Xavier Jimenez, Stacy D. Rodriguez, Alvaro Romero, Brittny N. Blakely, Sang-Yeon Cho, Charles Pelzman, Immo A. Hansen

**Affiliations:** 1 Department of Biology, New Mexico State University, Las Cruces, New Mexico, United States of America; 2 Department of Entomology, New Mexico State University, Las Cruces, New Mexico, United States of America; 3 Klipsch School of Electrical and Computer Engineering, New Mexico State University, Las Cruces, New Mexico, United States of America; 4 Institute of Applied Biosciences, New Mexico State University, Las Cruces, New Mexico, United States of America; North Carolina State University, UNITED STATES

## Abstract

The reliance on blood is a limiting factor for mass rearing of mosquitoes for Sterile-Insect-Technique (SIT) and other mosquito-based control strategies. To solve this problem, we have developed SkitoSnack, a formulated diet for *Aedes aegypti* (L) mosquitoes, as an alternative for vertebrate blood. Here we addressed the question if long-term yellow fever mosquito culture with SkitoSnack resulted in changed life history traits and fitness of the offspring compared to blood-raised mosquitoes. We also explored if SkitoSnack is suitable to raise Asian tiger mosquitos, *Aedes albopictus* (L.), and the human bed bug, *Cimex lectularius* (L). We measured life history traits for 30^th^ generation SkitoSnack-raised *Ae*. *aegypti* and 11^th^ generation SkitoSnack-raised *Ae*. *albopictus*, and compared them with control mosquitoes raised on blood only. We compared meal preference, flight performance, and reproductive fitness in *Ae*. *aegypti* raised on SkitoSnack or blood. We also offered SkitoSnack to bed bug nymphs. We found that long-term culture with SkitoSnack resulted in mosquitoes with similar life history traits compared to bovine blood-raised mosquitoes in both species we studied. Also, *Ae*. *aegypti* mosquitoes raised on SkitoSnack had similar flight performance compared to blood raised mosquitoes, were still strongly attracted by human smell and had equal mating success. Minimal feeding occurred in bed bugs. Our results suggest that long-term culture with the blood-meal replacement SkitoSnack results in healthy, fit mosquitoes. Therefore, artificial diets like SkitoSnack can be considered as a viable alternative for vertebrate blood in laboratory mosquito culture as well as for mosquito mass production for Sterile-Insect-Technique mosquito control interventions. SkitoSnack was not suitable to induce engorgement of bed bugs.

## Author summary

Novel, ‘green’ mosquito control strategies like Sterile-Insect-Technique, require the production of large numbers of mosquitoes from the targeted species. Females from these species require a meal of vertebrate blood to develop their eggs. Blood requires a cold chain for shipping and storage, has a short shelf life, and handling requires trained personnel. To address this problem, we have developed an artificial blood meal replacement called SkitoSnack that does not require cooling and has a much longer shelf life than blood. For this study we raised a colony of Yellow fever mosquitoes for 30 consecutive generations on SkitoSnack to test if long-term SkitoSnack culture affected mosquito fitness and life history traits. We found that 30th generation SkitoSnack-raised mosquitoes had equivalent egg numbers and hatch rates compared to blood-raised control mosquitoes and equal fitness. We found similar results for a closely related species, the Asian tiger mosquito, which we raised for 11 generations on SkitoSnack. We also found that SkitoSnack is not suitable to raise bed bugs. Together, our results show that SkitoSnack is a suitable meal for mass culture of yellow fever mosquitoes and Asian tiger mosquitoes.

## Introduction

Recent successful proof-of-principle experiments have shown that mosquito Sterile-Insect-Technique (SIT) has the potential to become a new tool to control populations of disease-transmitting mosquitoes [1, 2]. The yellow fever mosquito, *Ae*. *aegypti*, and the Asian tiger mosquito, *Ae*. *albopictus*, both vectors of important arboviral diseases in humans, have been targeted with SIT approaches [3–5]. *Wolbachia*-based dengue control efforts have been developed and supported by the World Mosquito Program [6, 7]. However, the broader implementation of mosquito SIT presents several challenges that needs to be addressed. For successful implementation of SIT, male mosquitoes must be raised in large numbers. Maintaining a mass mosquito culture in a laboratory setting requires a constant supply of vertebrate blood to produce viable eggs for experiments, trials, and SIT releases in the field. The current practice for blood feeding requires either the use of live animals or vertebrate blood that is offered via membrane feeders [8, 9]. Using live animals is heavily regulated in many countries and in all cases raises ethical concerns [10, 11]. There are also blood-quality and contamination issues. Additionally, vertebrate blood requires a cold chain for shipping and storage, has a short shelf life, and handling requires trained personnel. These factors increase the overall cost of SIT applications. Alternative approaches that reduce the cost of mass-rearing mosquitoes can significantly improve the SIT approach for controlling mosquito populations.

Artificial blood meal replacements for different species of mosquitoes have long since been the focus of scientific research. For an extensive discussion of problems and requirements of artificial diets in mosquito culture please see our review article published in 2016 [9]. In recent years, alternative artificial meals for *Aedes*, *Anopheles*, and *Culex* species have been reported [12–15]. In 2018, we developed and tested an artificial blood-meal replacement for *Ae*. *aegypti* called SkitoSnack [16]. SkitoSnack is composed of a bicarbonate buffer, ATP as a phagostimulant, bovine serum albumin, chicken yolk as primary nutrient source, and bovine hemoglobin as an iron source. Our previous study demonstrated that feeding SkitoSnack to female *Ae*. *aegypti* resulted in similar engorgement rates, egg numbers and hatch rates compared to blood fed mosquitoes. While we only found a few differences in the metabolome of eggs derived from SkitoSnack or blood feeding, the midgut microbiome was notably changed between females fed on the two different diets. While we calculated the cost of SkitoSnack to be similar to commercially purchased vertebrate blood, the long shelf life of SkitoSnack stored as a powder makes it a true alternative to vertebrate blood.

In this study, we compared the life history traits and fitness of *Ae*. *aegypti* mosquitoes raised for over 30 generations and *Ae*. *albopictus* mosquitoes raised for 11 generations on SkitoSnack compared to blood-raised mosquitoes. Our results support the conclusion that SkitoSnack can be used for mosquito mass culture to produce competitive mosquito males for SIT and other mosquito-based control strategies. We also found that SkitoSnack is not suitable for bed bug culture and will require further modifications.

## Materials & methods

### Mosquito strains

All mosquito strains used in this study (**see Table 1**) were obtained through BEI Resources except the *Ae*. *aegypti* UGAL strain that was contributed by Alexander Raikhel, University of California Riverside. All strains were propagated in the laboratory using bovine blood for a minimum of five generations before the experiments were conducted. Starting from the same stock, we have propagated parallel cultures of UGAL mosquitoes, one fed on bovine blood, the other one on SkitoSnack since 2017 [16]. Similarly, we raised parallel cultures of *Ae*. *albopictus*, Gainesville strain, fed on the two different diets, for eleven generations.

**Table 1:** Summary of mosquito strains and species used in this study.

Species	Strain	MR4 order number	Contributor
*Aedes aegypti*	UGAL	n/a	Raikhel laboratory
*Aedes aegypti*	ROCK	MRA-734	David W. Severson
*Aedes aegypti*	Black Eye Liverpool	NR-48921	Filariasis Research Reagent Resource Center
*Aedes albopictus*	Gainesville	MRA-804	Sandra A. Allan
*Aedes albopictus*	ATM-NJ95	NR-48979	Centers for Disease Control and Prevention

The strains used in our study are long-time laboratory strains. ROCK, short for Rockefeller, was approximately established in 1881. The Liverpool strain dates back to the 1930s, while the UGAL strain, short for University of Georgia Lab strain, is undocumented and was presumably established in the 1970s [17].

### Mosquito culture

Mosquito rearing was carried out according to Kandel et al., 2019 [18]. We standardized the rearing conditions of the different batches of mosquitoes to avoid larval overcrowding in each rearing pan. Adult mosquitoes were held in BugDorm 1 cages 30 × 30 × 30 cm (#1452, BioQuip Products, Rancho Dominguez, CA) with access to water and a separate bottle with 20% sucrose solution. The BugDorm cages were placed inside a dedicated climate-controlled culture room at 27°C, 16:8 hours light/dark cycle, and 80% relative humidity.

### Mosquito feeding assay

Mosquito feeding assays were performed as described in Gonzales et al., 2015 [8] with minor modifications. Adult mosquitoes, one-week post emergence, were used for all the feeding experiments. The mosquitoes were sugar-starved for at least 16 hours before feeding. For each replicate, 15–40 female mosquitoes were collected from a BugDorm cage with an battery-powered aspirator (# RHM200, Gempler’s, Janesville, WI) and transferred to a new BugDorm cage. Then, 3 mL of either SkitoSnack or defibrinated bovine blood (Hemostat, Dixon, CA) was offered for 60 minutes using a water-heated glass feeder with 4 cm diameter [19]. The system consisted of a custom-made water-jacketed glass feeder with a Parafilm membrane (Sigma Aldrich, St. Louis, MO) stretched across the bottom through which the insects fed. After 60 minutes, all mosquitoes were collected and anesthetized with CO_2_. Engorgement was determined by visual inspection with a Stereo microscope (Olympus, SZ-STS, Japan). Mosquitoes that presented red color from blood or blue or green food color (Limino food color, Universal Product Code #682559432732, purchased from Amazon) from SkitoSnack in their abdomens were counted as engorged. Engorgement rates were calculated by dividing the number of fed mosquitoes over total number of mosquitoes in each respective trial.

### Egg production assay

Egg-laying chambers for individual mosquito was constructed as described by Gonzales et al., 2018 [16]. Briefly, the bottom of a 50 mL conical Falcon tube was punctured three times with a 16-gauge needle. A cotton ball was inserted in the Falcon tube and covered with a circular germination paper. An individual engorged mosquito was selected and transferred into a dry egg laying chamber. After 24 hours, the bottoms of the egg laying chambers were submerged in water to create a wet substrate suitable for mosquito egg deposition. After 6 days, the germination paper on top of the substrate was taken out and egg numbers were determined using a stereo microscope and a handheld tally counter.

### Egg viability assay

The germination paper circles with mosquito eggs were air dried in Petri dishes (VWR, Radnor, PA) and stored for 1 week in an incubator at 27°C, 70% humidity. For each biological replicate, 100 eggs were selected and transferred using a paint brush into a shallow pan (28 cm x 32 cm x 4 cm) with 1.5 liters of water. Each pan was provided with 1 pellet of Special Kitty cat food. The number of live larvae was determined after four days by visual inspection. It should be noted that the results of this assay integrated hatch rates and larval mortality during the first four days.

### Irradiation/Longevity assay

To understand the impact of long-term culture with SkitoSnack on mosquitoes, the longevity of male *Ae*. *aegypti* (UGAL strain) raised for 22 consecutive generations on SkitoSnack was evaluated. Mosquitoes raised on bovine blood were reared in parallel and used as a control group. Mosquitoes, 24 hours post emergence, were aspirated from BugDorm cages, and anesthetized on ice for 1 minute. The males were separated from females using a bird feather and transferred to a 35 × 10 mm petri dish (25 per dish). The covered petri dishes were placed on the rotating platform of an X-ray irradiator (Faxitron, Tucson, AZ) and irradiated with a dosage of 50 Gy. Control mosquitoes received an identical mock treatment with the irradiator switched off. The males were then placed in BugDorm cages and incubated at 27°C, 16 : 8 light/dark cycle and 70% relative humidity. Mosquitoes were provided 50 mL flasks with cotton wicks of 20% sucrose solution and de-ionized (DI) water that were replaced every day. Mortality was observed daily until all mosquitoes were dead.

### Meal preference assay

This assay was developed to determine if mosquito females showed preference for blood vs. SkitoSnack meals. *Ae*. *aegypti* (UGAL strain) mosquitoes, raised on SkitoSnack for 30 consecutive generations were used for this experiment and compared with mosquitoes raised on bovine blood. Eggs of bovine blood-raised and SkitoSnack-raised mosquitoes were vacuumed hatched for 15 minutes in 100 mL of distilled water. The emerged larvae were transferred into a 45 × 30 × 6 cm shallow pan with 2 L of water and given access to five pellets of cat food. The next day, the larvae were separated into four 45 × 30 × 6 cm shallow pans each containing 500 larvae and were given five pellets of cat food. The pans were monitored daily and cat food was added as needed. Pupae from each pan were transferred into an 11.5 cm diameter × 3.5 cm deep round plastic cup, filled with distilled water, and placed in BugDorm cages for emergence. Nine experimental replicates, each with 20–40 randomly selected mosquitos from four different BugDorm cages were used for this feeding assay. Adult female mosquitos, one-week post emergence, were starved for a minimum of 16 hours and transferred into BugDorm cages using a battery-powered aspirator. SkitoSnack and bovine blood were offered in glass membrane feeders side by side in the middle of the cages for 1 hour. Food dyes mixed into SkitoSnack (Limino food color, Universal Product Code #682559432732, Amazon), were used to identify mosquitoes that were engorged on the artificial meal. After 1 hour, the mosquitoes were aspirated out of the BugDorm cage, anesthetized on ice and the number of mosquitoes fed on each of the two different meals was determined based on the red color of the ingested blood or the blue/green color of SkitoSnack.

### Host attraction bioassay

A Y-tube olfactometer bioassay was conducted to determine the attraction rate of SkitoSnack-raised mosquitoes towards a human host. *Ae*. *aegypti* (UGAL strain), raised on SkitoSnack for 22 consecutive generations were used for this experiment and compared with mosquitoes raised on bovine blood. The construction of the Y-tube olfactometer followed WHO guidelines [20, 21]. This bioassay protocol is based on a previous study described by Mitra et al., 2020 [22]. Briefly, a volunteer’s hand was placed in the hand chamber of the Y-tube, and the other chamber was left empty. About 20–30 adult female mosquitoes were aspirated into the holding chamber. After 1 minute of acclimation in the tube, the door of the holding chamber was opened allowing mosquitoes to fly either towards the volunteer hand or the empty chamber. After 2 minutes 45 seconds, all the doors were shut, and the number of mosquitoes present in each chamber were counted. The percent attraction was calculated by dividing the number of mosquitoes in the hand chamber by the total number of mosquitoes released inside the Y-tube.

### Mosquito life history measurements

Eggs from bovine blood-raised and SkitoSnack-raised mosquitoes were vacuum-hatched for 15 minutes in 100 ml of distilled water. Mosquito larval culture was conducted as described above using a standardized protocol to ensure all groups were raised using similar treatment. Mosquitos, 7–10 days post emergence, were starved for 16 hours and random samples of females and males were taken from each BugDorm cage. The weight of individual mosquito was determined using an XPR micro balance (Mettler Toledo, Columbus, OH). The wing lengths of individual mosquito was determined using a stereo microscope and a micro scale (S1 stage micrometer, PYSER-SGI, United Kingdom).

### Flight mill assay

**Fig 1:**
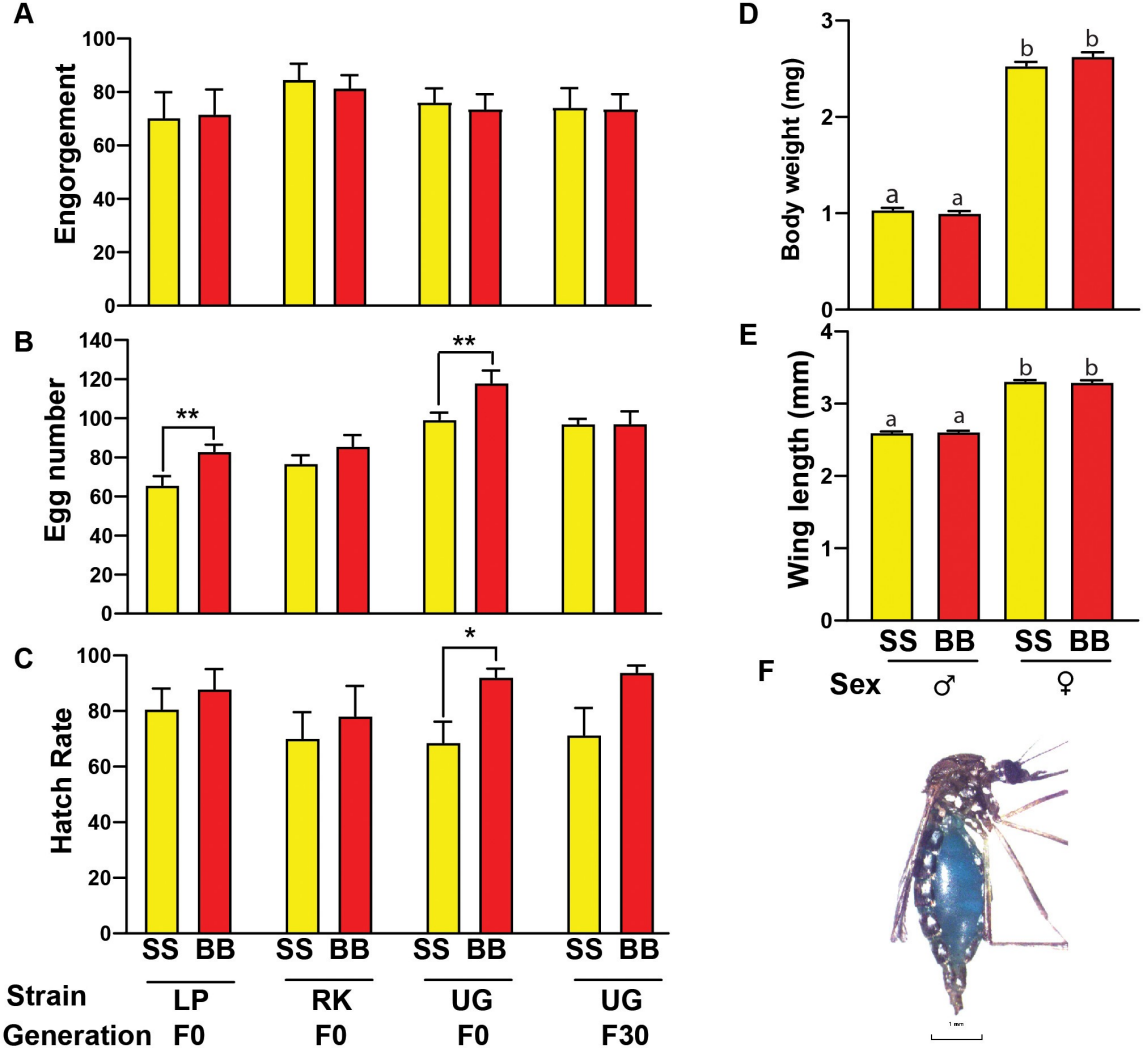
Reproductive Performance of SkitoSnack-raised *Aedes aegypti*. **—(A)** Percent engorgement on SkitoSnack (SS) and bovine blood (BB) in different strains. **(B)** Average number of eggs laid per individual female comparison between SkitoSnack and bovine blood in different strains. **(C)** Egg hatch rate comparison between SkitoSnack and bovine blood-fed females of different strains. **(D)** The average weight comparison between 30^th^ generation bovine blood and SkitoSnack-raised mosquitoes. **(E)** The average wing length comparison between 24^th^ generation bovine blood and SkitoSnack raised mosquitoes. **(F)** *Ae*. *aegypti* engorged on SkitoSnack. The blue color is due to the added food color in the meal.

To test mosquito fitness, a flight mill (**Fig 1A**) was constructed based on an earlier publication [23]. A detailed protocol for its construction and the flight experiments is provided in

**S1 File**.

### Mating competitiveness assay

Stable isotope labeling was performed as described by Helinski et al., 2012 [24] with some minor modifications. We labeled four groups of male *Ae*. *aegypti* (UGAL strain) with two different stable isotopes added directly to the water during their larval development. For ^15^N labeling, 200 μL of a 10% (w/v) ammonium sulfate stock solution (5 atom % ^15^N, #IN 5072, Berry & Associates/ICON Isotopes, Dexter, MI) was added to 200 ml DI water. For ^13^C labeling, 200 μL of a 17% glycerol stock solution (99 atom % ^13^C, #489476, Sigma Aldrich, St. Louis, MO) was added to 200 mL of DI water. Larval and pupal development occurred in 500 mL soup cups (Amazon). SkitoSnack-raised and blood-raised males were labeled in separate groups with both stable isotopes. Emerging males were separated and stored in BugDorm cages for at least three days with access to DI water and 20% sucrose. Unlabeled female virgins were collected after emergence and kept in a separate BugDorm cage. For the mating competition, a SkitoSnack-raised male labeled with one stable isotope and a blood raised male, labeled with the other stable isotope, were combined with an unlabeled virgin female in a BugDorm cage (17.5 x 17.5 x 17.5 cm) and allowed to mate for 24 hours. After 24 hours, the females were transferred into a 1.5 mL Eppendorf tube, frozen, and dried at 60°C overnight. The samples were sent to UNM Center for Stable Isotopes for Isotope Ratio Mass Spectrometry analysis (IR-MS) [25]. Paternity was established by either an increase in the d ^15^N value or a decrease in the d ^13^C value compared with unlabeled blood raised-females that were mated with unlabeled males.

### Bed bug feeding experiments

*Cimex lectularius*, Harlan strain, were used for these experiments. Groups of twenty 5^th^ instar female nymphs were separated from the male nymphs, seven days after emergence. The nymphs were offered SkitoSnack, rabbit blood (Hemostat, Dixon, CA) as a positive control, or PBS buffer with 3 mM ATP as a negative control. Four biological replicates were performed for each treatment group. Meals were offered at 37°C for 1 hour using glass feeders with artificial membranes. Percent engorgement was calculated as the number of engorged bed bugs divided by the total number of bed bugs in each replicate. All engorged bed bugs were kept in separate round plastic jars (Consolidated Plastics, Stow, OH) with 300 μm plankton mesh (Bioquip, Rancho Dominguez, CA) on the bottom. Percent molting was determined as the number of bugs that molted within seven days to the adult stage divided by the total numbers of bed bugs in each replicate. Mortality was determined seven days after feeding.

### Statistical analysis

All statistical tests were performed using Prism8 (GraphPad software, San Diego, CA). P-values less than 0.05 were considered significant. The distribution of the data was analyzed using Shapiro-Wilk test. If the data were normally distributed, means between two groups were compared using a student’s *t*-test. If the data were not normally distributed, a Mann‐Whitney U test was used to evaluate the statistical significance. For comparison between multiple groups of non-normally distributed data, a Kruskal-Wallis test was used followed by Dunn's post hoc test for multiple comparisons. For normally distributed data a one-way ANOVA was used followed by Tukey’s post hoc test for multiple comparisons. The survival data were analyzed using a Kaplan-Meier test to identify significant differences between mortality curves.

## Results

### Female reproductive performance after feeding SkitoSnack or bovine blood to *Aedes aegypti*

**Fig 1** shows the reproductive performance of females from three different strains of *Aedes aegypti* that were offered SkitoSnack compared with egg numbers from females that were offered bovine blood. We also show these data for a strain (UGAL) that was raised exclusively on SkitoSnack for 30 generations. We found that engorgement rates were not significantly different between all groups we tested (Mann-Whitney U test) (**Fig 1A**).

We found significant differences in egg numbers between blood-fed females and mosquitoes fed on SkitoSnack in the Liverpool and UGAL F0 strains. We did not find a significant difference in the number of eggs deposited between ROCK females and the UGAL F30 females fed on blood and those that were fed on SkitoSnack (Mann-Whitney U test) (**Fig 1B**).

Hatch rates were found to be significantly higher between female UGAL F0 fed on blood and those that were fed on SkitoSnack. No significant difference was found in the other strains we analyzed (Mann-Whitney U test) (**Fig 1C**).

**Fig 1D** and **1E** show the average body weight and wing length of UGAL strain mosquitoes that were raised for 30 generations on SkitoSnack and control mosquitoes raised on bovine blood. While we found significant differences in both body weight and wing length between males and females, no significant differences were found between SkitoSnack and bovine blood-raised mosquitoes.

Mosquito engorgement was confirmed by visual inspection (**Fig 1F**).

The results of our statistical analysis are shown in **S1 File**.

### Female reproduction after feeding SkitoSnack or bovine blood to *Aedes albopictus*

**Fig 2:**
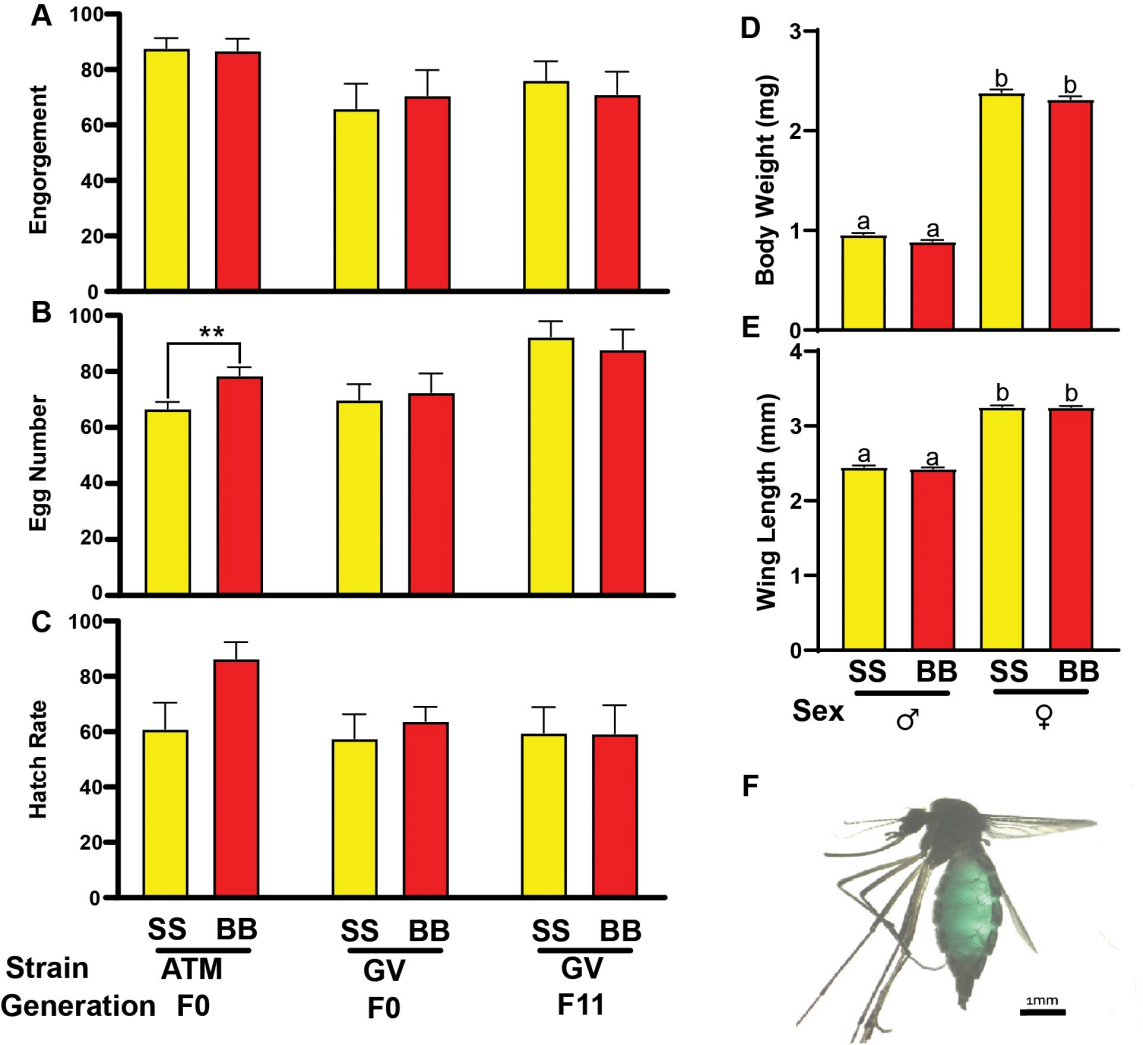
Female reproductive performance after feeding SkitoSnack or bovine blood to *Aedes albopictus*. ***–*(A)** Percent engorgement on SkitoSnack (SS) and bovine blood (BB) in different strains. A Kruskal -Wallis H test showed that there was not a statistically significant difference in engorgement rate between treatment groups, (H = 8.69, p = 0.1221) **(B)** Average number of eggs laid per individual female comparison between SkitoSnack and bovine blood in different strains. **(C)** Egg hatch rate comparison between SkitoSnack and bovine blood in different strains. **(D)** The average weight comparison between 11^th^ generation bovine blood and SkitoSnack-raised mosquitoes. Different letters indicate significant difference at P < 0.05. **(E)** The average wing length comparison between 11^th^ generation bovine blood and SkitoSnack raised mosquitoes. Different letters indicate significant difference at P < 0.05. **(F)** *Ae*. *albopictus* engorged on a SkitoSnack, the green color is due to the added food color in the meal.

We offered SkitoSnack and bovine blood to two different strains of *Ae*. *albopictus*: Gainesville and ATMN, and a Gainesville mosquito strain that was raised for 11 consecutive generations on SkitoSnack. Engorgement rates were not significantly different between our experimental groups (Mann-Whitney U test) (**Fig 2A**).

ATM-NJ95 females fed on bovine blood produced more eggs than females fed on SkitoSnack. No significant differences in clutch size were found between SkitoSnack and bovine blood fed females in both F0 and F11 Gainesville-strain (Mann-Whitney U test) (**Fig 2B**).

Hatch rates were not significantly different between all experimental groups (Mann-Whitney U test) (**Fig 2C**).

**Fig 2D** and **2E** show the average body weight and wing length of Gainesville-strain *Aedes albopictus* mosquitoes of both sexes that were raised for 11 generations on SkitoSnack and control raised on bovine blood. While we found significant differences in body weight and wing length between males and females, there were no significant differences between SkitoSnack and bovine blood-raised mosquitoes (Kruskal-Wallis test followed by Dunn's multiple comparisons test).

Mosquito engorgement was confirmed by visual inspection (**Fig 2F**).

The results of our statistical analysis are shown in **S1 File**.

### Meal preference of female *Aedes aegypti*

**Fig 3:**
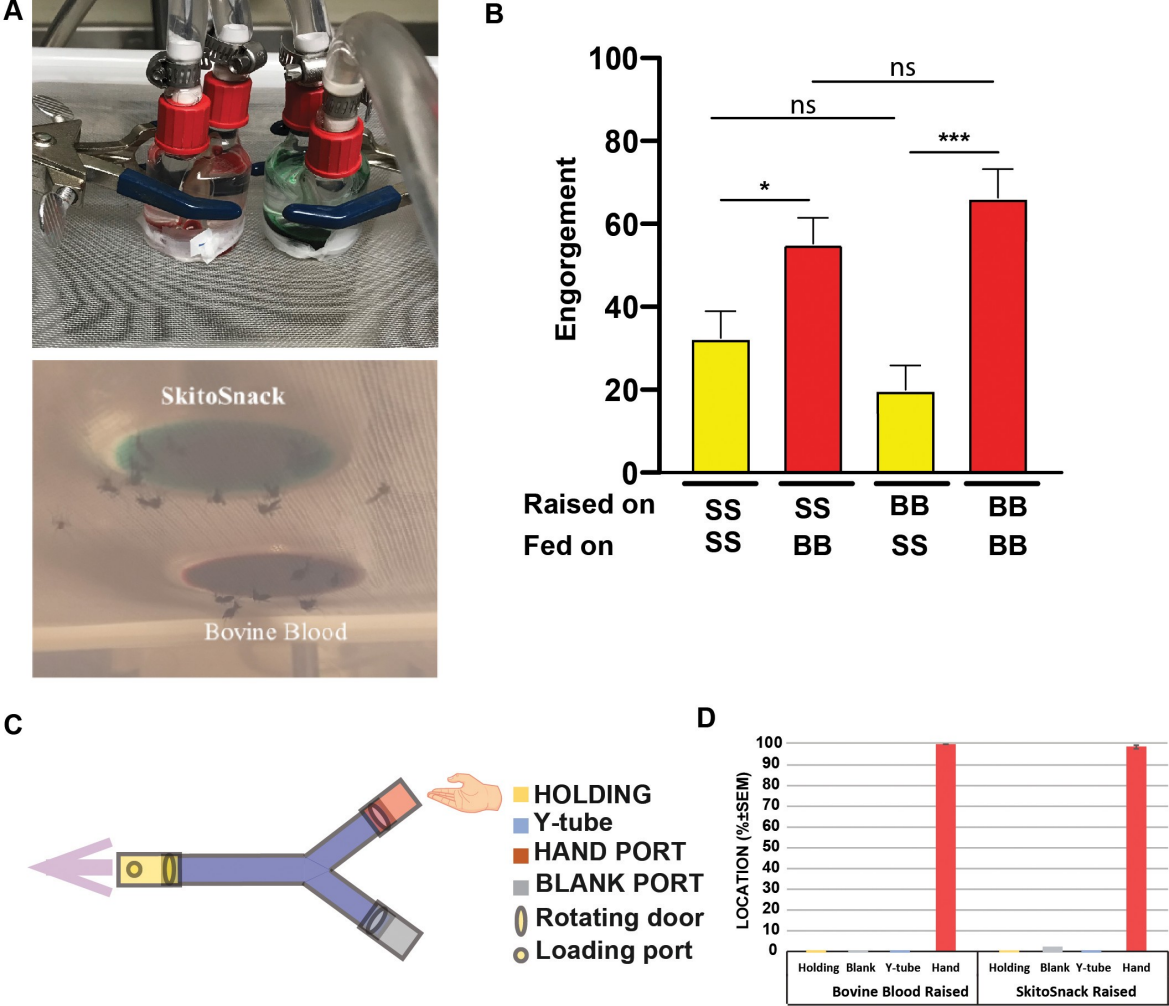
Mosquito meal preference. **–(A, upper panel)** Experimental setup. The left feeder contains bovine blood, the right SkitoSnack with added green food color. **(A, lower panel)** The same setup seen from below. **(B)** Percent engorgement on SkitoSnack (SS) or bovine blood (BB) of mosquitoes raised on these meals. Ns–indicates no significant difference at P < 0.05. **(C)** Schematic diagram of the experimental setup for Y-tube olfactometer bioassay [21]. The arrow indicates the direction of the air flow. **(D)** Percent attraction to a human hand in a Y-tube attraction assay of SkitoSnack (SS) raised or bovine blood (BB) raised females.

**Fig 3** shows the preference of different groups of female *Aedes aegypti*, that were offered SkitoSnack and bovine blood at the same time (**Fig 3A and 3B**). Mosquitoes from both, the SkitoSnack-raised and the bovine blood-raised groups preferred bovine blood over SkitoSnack when given the choice (Mann-Whitney U test).

### Female attraction to a human host

We compared the attraction to a human host between SkitoSnack-raised and bovine blood-raised mosquitoes using Y-tube attraction assays (**Fig 3C**). There was no significant difference between the attraction rates measured for SkitoSnack-raised mosquitoes and mosquitoes raised on bovine blood (**Fig 3D**) (Mann-Whitney U test, U = 6, N1 = 4, N2 = 4, P>0.999, two-tailed).

### Flight performance of male mosquitoes raised on SkitoSnack

**Fig 4:**
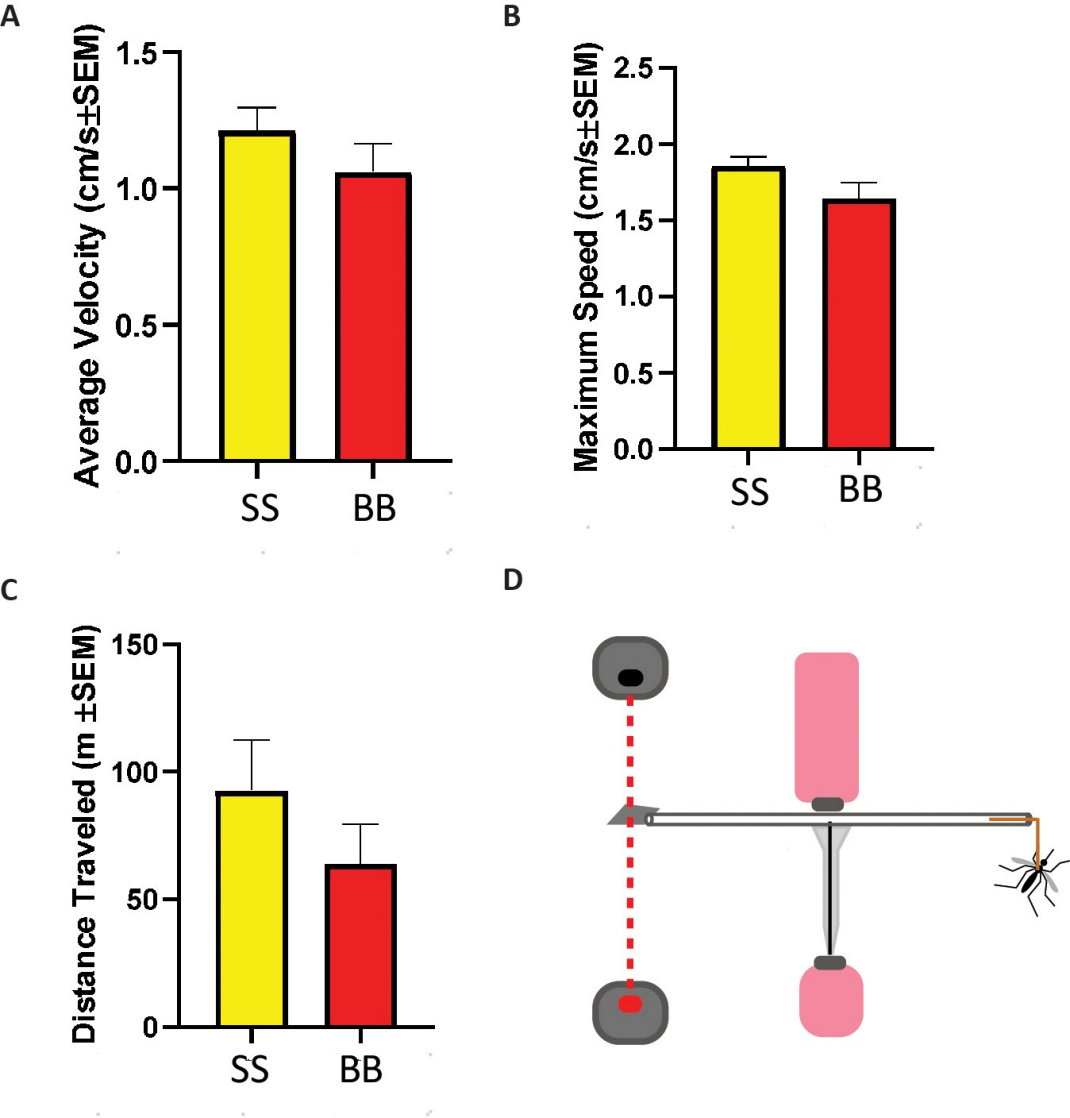
Flight performance of mosquitoes on the flight mill. **(A)** Average velocity of *Aedes aegypti* males raised on SkitoSnack (SS) or bovine blood (BB). **(B)** Maximum speed of *Aedes aegypti* males raised on SkitoSnack (SS) or bovine blood (BB). **(C)** Average distance traveled of *Aedes aegypti* males raised on SkitoSnack (SS) or bovine blood (BB). **(D)** Schematic diagram of mosquito flight mill. For details please see supplemental files.

We used a custom-built flight mill to measure flight parameters in *Aedes aegypti* mosquitoes from a strain raised for 30 generations on SkitoSnack and control mosquitoes from the same stock raised for 30 generations on bovine blood. **Fig 4** shows average velocity, maximum speed, and distance traveled for groups of males. We found no significant differences in these parameters between males from groups raised on SkitoSnack or bovine blood.

The results of our statistical analysis are shown in **S1 File**.

### Longevity of irradiated males

**Fig 5:**
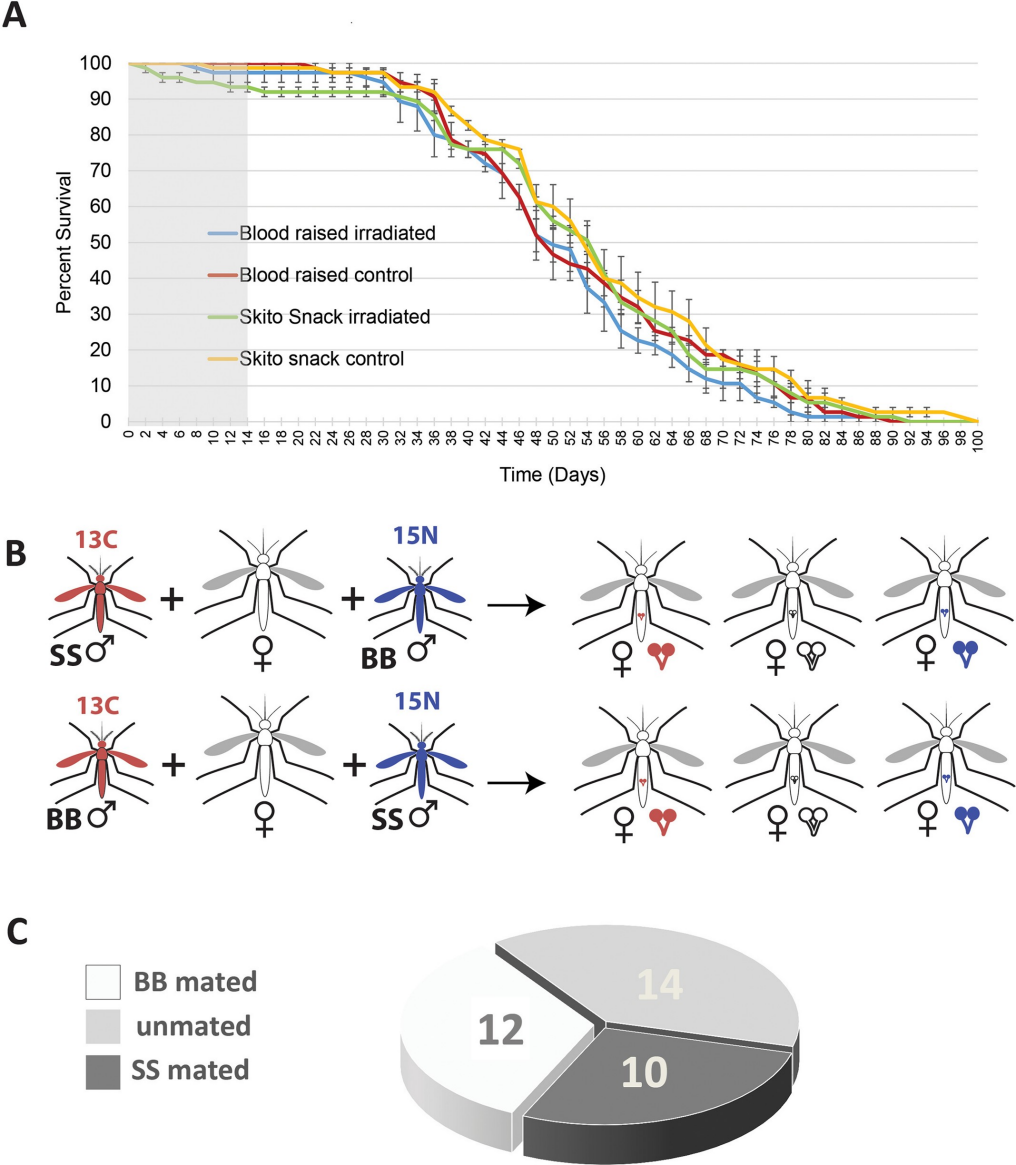
Fitness of SkitoSnack-raised mosquito males compared to bovine blood-raised males. **(A)** Survival curve of mosquito males from SkitoSnack culture and bovine blood culture with and without radiation treatment. The grey box marks the first fourteen days after the start of the experiment that were analyzed as an individual dataset. **(B)** Experimental setup scheme for the mating competition assay. To avoid a labeling bias, males from both diet groups, SkitoSnack (SS), and bovine blood (BB) were labeled with each stable isotope. On the right side, the three different outcomes we observed are shown. Half of the matings were set up as shown in the upper panel, the other half were set up as shown in the lower panel. **(C)** Paternity test results. Shown is the number of females that mated with a male from a specific diet group and the number of females that did not mate. No double matings were observed.

We compared the longevity of male *Ae*. *aegypti* UGAL mosquitoes that were raised on SkitoSnack for 22 generations with males raised on bovine blood with and without radiation treatment. We found no significant difference between all four groups (**Fig 5A**) (Kaplan-Meier Test, bovine blood irradiated vs. SkitoSnack irradiated (p = 0.193), bovine blood irradiated vs. bovine blood control (p = 0.315), SkitoSnack irradiated vs. SkitoSnack control (p = 0.353), and SkitoSnack control vs. bovine blood control (p = 0.249). We then analyzed survival during the first 14 days after irradiation in all four groups. We found a significant increase in mortality in irradiated males from SkitoSnack culture when compared to the other groups. The results of our statistical analysis are shown in **S1 File.**

### Mating competitiveness of SkitoSnack-raised *Aedes aegypti* males

We performed mating competition experiments as shown in **Fig 5B** with subsequent stable isotope-based paternity testing. Out of 36 females that we analyzed, 14 females did not mate, 12 females mated with a blood-raised male, and 10 females mated with a SkitoSnack-raised male (**Fig 5C**).

### Bedbug engorgement and development after feeding SkitoSnack

**Fig 6:**
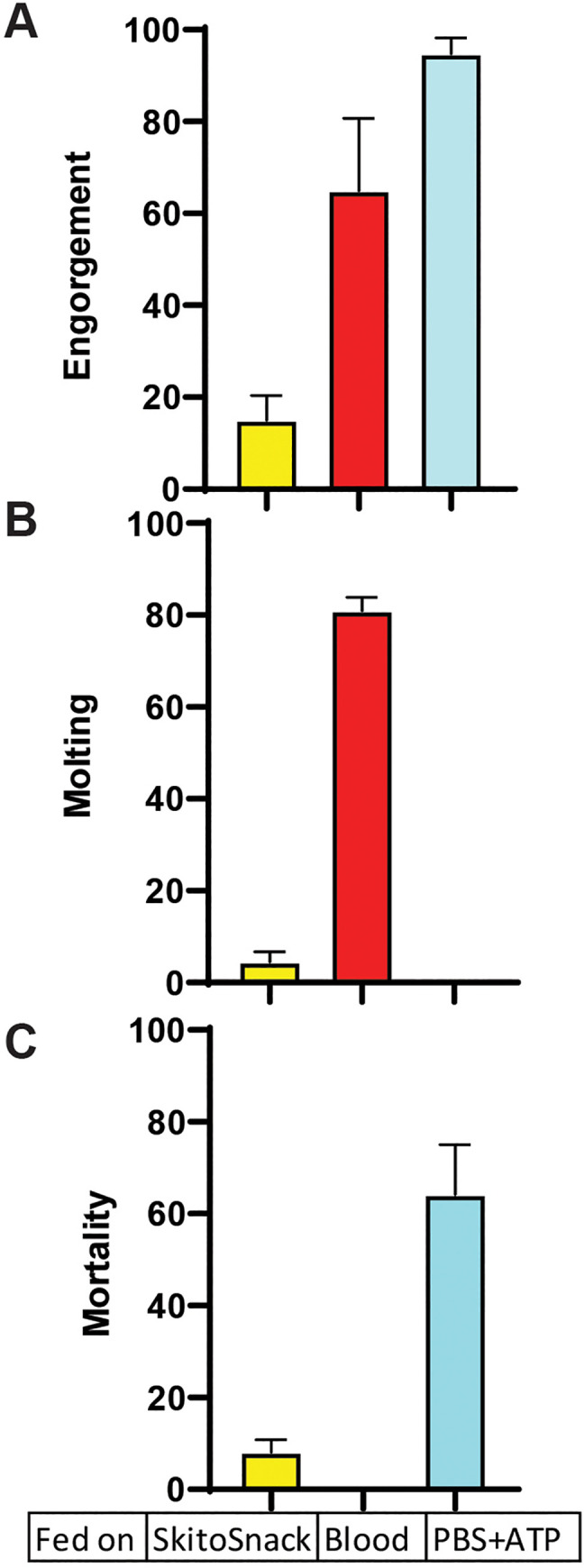
Bedbug development after feeding SkitoSnack. Shown are the **(A)** percent engorgement, **(B)** percent of bug nymphs that molted into adults, and **(C)** percent mortality, seven days after feeding SkitoSnack, bovine blood (Blood), or a PBS solution with 3 mM ATP.

We found that only a small percentage of 5^th^ instar bedbug nymphs engorged on SkitoSnack compared to rabbit blood or a PBS solution with ATP (**Fig 6**). While 80% of the blood-fed nymphs molted into adults, only 3% of the SkitoSnack-fed nymphs molted into adults. Mortality after seven days was 6% with SkitoSnack, 0% with blood, and 62% with the PBS solution.

## Discussion

Most disease-transmitting mosquito females are strongly anautogenous; they require a meal of vertebrate blood in order to develop eggs [26]. Vitellogenesis, the production of yolk proteins and their deposition in the eggs, depends on nutrient amino acids that are derived from blood proteins [27, 28]. Therefore, culture of anautogenous mosquitoes in laboratories or dedicated rearing facilities depends on a constant supply of vertebrate blood. Finding a replacement for vertebrate blood has been the focus of research for some time with varying degrees of success [9, 13–15].

SkitoSnack was developed by performing a number of trial and error experiments in order to optimize engorgement rates, egg numbers, and most importantly egg hatch rates for *Ae*. *aegypti* [16]. While the outcomes of that study were very positive, the question remaining was if long-term mosquito culture using this artificial blood meal replacement would result in changes in mosquito life history traits, their behavior and fitness. The present study provides evidence that mosquito culture using SkitoSnack produces mosquitoes with equal life history traits and fitness than mosquito culture with vertebrate blood.

We compared reproductive outcomes for three different laboratory strains of *Ae*. *aegypti*. We did not find any significant differences between these three strains in the percentage of mosquitoes that engorged when offered blood or SkitoSnack (**Fig 1A**). However, egg numbers produced after feeding different diets varied between the different strains (**Fig 1B**). While egg numbers are obviously an important parameter to determine the success of an artificial blood meal replacement, egg hatch rates are often a critical problem [9]. For example, in an earlier study we found that a simple BSA solution results in high egg numbers when fed to female *Aedes aegypti*. The eggs produced had very low hatch rates [8]. In the current study, egg hatch rates were not significantly different between females from the Liverpool and ROCK strains when fed on either diet. Only eggs from the UGAL strain showed slightly increased hatch rates when females were fed on blood (**Fig 1C**). Together, these results show that feeding SkitoSnack to female *Ae*. *aegypti* resulted in the development of viable eggs at numbers comparable to those developed by blood-fed mosquitoes.

An important consideration that needs to be addressed before SkitoSnack can be established as a blood meal replacement is if long-term mosquito culture with SkitoSnack affects the life history traits, behavior, and fitness of mosquitoes. To address this question, we raised a strain of *Ae*. *aegypti* UGAL mosquitoes exclusively on SkitoSnack for 30 generations. A control strain of UGAL mosquitoes was raised in parallel on blood only. Both of these strains showed very similar reproductive performance and life history traits (Fig 1).

As mentioned above, SkitoSnack was developed using *Ae*. *aegypti* as test subjects. The Asian tiger mosquito, *Ae*. *albopictus*, is a closely related species and transmits many of the same diseases [29–31]. Our results (**Fig 2**) suggest that SkitoSnack is also a suitable blood meal replacement for *Ae*. *albopictus*. Interestingly, we found that eleventh generation SkitoSnack females produced more eggs than females from the blood-raised control group when both were fed SkitoSnack. These observations suggest that SkitoSnack is suitable for long term culture of *Ae*. *albopictus* and that the strain raised on SkitoSnack over eleven generations has adapted to this meal.

Host preference in blood feeding mosquitoes is a well-studied phenomenon [32–34]. One question, which regularly arises concerning artificial blood meal replacements is if mosquitoes prefer blood or an artificial meal if given a choice. In our feeding system consisting of water-heated glass feeders with Parafilm membranes, heat is thought to be the principal cue that attracts mosquitoes to the feeders and initiates blood feeding behavior [35]. Therefore, our initial hypothesis was that there would be no feeding preference for a specific diet if both are offered simultaneously in glass feeders at the same temperature. Surprisingly, blood-raised control mosquitoes showed a significant preference for blood over SkitoSnack when both were offered at the same time (**Fig 3A–3C**). SkitoSnack-raised mosquitoes also preferred blood over SkitoSnack in this assay. These results suggest that blood and SkitoSnack emit different olfactory cues through the Parafilm membrane to the host-seeking females when offered in a glass feeder. An alternative hypothesis is that SkitoSnack does not exhibit the same phagostimulatory effect as vertebrate blood. SkitoSnack contains 3 mM of ATP, a potent phagostimulant for mosquitoes and other blood-sucking arthropods [9, 36], but other components might act as feeding deterrents that cause probing mosquito females to reject this meal. Further work is necessary to identify the cues, responsible for this preference for vertebrate blood. After more than 30 generations of being fed exclusively on SkitoSnack, mosquito females were also still strongly attracted by human odor (**Fig 3D**).

One major concern regarding long-term mosquito culture with SkitoSnack is that missing micronutrients or vitamins would lead to a decrease in fitness over time. To address this concern, we used a flight mill to compare several flight fitness parameters of SkitoSnack-raised and bovine blood-raised mosquitoes [23]. Flight mill have long since been used to study insect fitness [37–39]. We focused on male mosquitoes in our experiments because they are more likely to be used in mosquito-based control efforts. Using this assay, we show for the first time, that culture with SkitoSnack results in males with similar flight performance as males from blood-based culture (**Fig 4**).

Mosquito longevity is an important factor influencing vectorial capacity in females [40]. In males it is an important consideration for the planning of SIT interventions [1, 41, 42]. We did not find significant differences in the long-term survival curves of *Ae*. *aegypti* males from SkitoSnack culture and blood culture in both, radio-sterilized and un-irradiated control mosquitoes (**Fig 5A**). However, when we analyzed the mortality data during the first two weeks after irradiation and compared them to un-irradiated control mosquitoes, we found a small but significant increase in mortality in irradiated males from SkitoSnack culture when compared to the other groups.

Mating success in the field is critical for the success of SIT interventions [43–45]. Our results showed that SkitoSnack-raised males had equal mating success as blood-raised males in cage experiments (**Fig 5B & 5C**). We did not observe a single case of polyandry as has been shown in other cage experiments [24]. This might be due to the shorter mating time and the smaller number of matings in our experiment. It must be noted that the conditions of this test are highly artificial and don’t reflect field conditions that a male might encounter during a SIT intervention. More extensive cage and field tests with higher sample sizes are necessary to define the reproductive success of SkitoSnack- raised males when they compete with wild males in the field.

Encouraged by these very positive outcomes we had in our mosquito experiments, we next tested if SkitoSnack is also suitable for culture of a hemimetabolous insect, the common bed bug, *Cimex lectularius* (**Fig 6**). From our results, it is apparent that SkitoSnack is lacking one or more ingredients to allow bed bug development. More studies are necessary to identify these components.

In summary, replacing vertebrate blood is not only desirable from a practical or financial point of view, but also from an ethical one, since it will reduce the number of laboratory animals used as blood sources. Here we show for the first time that an artificial blood meal replacement can replace vertebrate blood in mosquito long-term mass culture of *Ae*. *aegypti* without major changes in their reproductive output or fitness. SkitoSnack powder has a basically unlimited shelf life, does not require cold storage, and the feeding solution can be prepared in small batches.

Future projects will focus on the development of suitable artificial diets for other species of blood-sucking arthropods.

## Supporting information

S1 File This Excel spread sheet contains the raw data and results of the statistical test that we performed for the individual figures. (XLSX)

S2 File This word file contains protocols for flight mill construction and our flight protocol. (DOCX)

S3 File This video file shows the rotation of the flight mill arm by an attached mosquito. (MP4)
