# Supplementary material for: Long-Term Mosquito culture with SkitoSnack, an artificial blood meal replacement
Source: PLoS Negl Trop Dis. 2020 Sep 17;14(9):e0008591. doi: 10.1371/journal.pntd.0008591 (PMC7523998; doi:10.1371/journal.pntd.0008591)
Supplement: S2 File — (DOCX) [file pntd.0008591.s002.docx]

**Flight mill construction and experimental protocol:**

**Construction of the acrylic plastic Structure:** A flight mill was constructed as previously described (Attisano et al., 2015). Briefly, 3 mm thick acrylic Plexiglass pieces were cut with a Dremel® power tool and buffed with sandpaper. Each vertical wall was 9 × 9 cm, and the horizontal floor and roof were 9 × 20 cm. The Plexiglas box was assembled using hot glue as seen in Figure 1.

**Construction of the rotating/pivoting arm:** Lever arms made from hypodermic steel needle have been previously used to track movement in different insect models (Attisano et al., 2015, Naranjo, 2019, Ribak et al., 2017). Application of similar rotating arms with mosquitoes failed to rotate the hypodermic steel needle due to small body size and heavy weight. Therefore we designed and implemented two different types of glass capillaries as lever arms, thin-walled tubes weighing 0.12 g for males (world precision Instruments, Florida, catalogue # TWT100-6) and a thicker walled tubes weighing 0.22 g for females (World Precision Instruments, Florida, catalogue # 1B100-6) as highlighted in Figure 1. The lever arm was attached to a 10 µl pipetting tip with a #3 entomological metal pin (Austerlitz insect pins, size 3) placed inside to provide a pivot. This assembly was placed between two 10 × 4 mm N42 neodymium magnets held on the center of the floor and ceiling of acrylic sheets. The magnets were attached to rubber tubing (width of 1.5 cm, length of 2 cm, and internal diameter of 0.7 cm) with the help of hot glue gun making sure the top and bottom magnets were aligned. The purpose of the magnets was to place the entomological pin in the vertical position allowing the glass capillary arms to revolve around its axis to track the movement of attached mosquitoes with little resistance.

**Infra-red sensor set up:** Two infra-red (IR) beam break sensors were attached to the internal side of the top and bottom of the acrylic sheets as seen in Figure 1. Passing of the aluminum foil attached at the end of a glass capillary breaks the beam path as the attached mosquito flies and rotates the arm. The IR-sensor was connected to an Arduino microcontroller to detect the break in the beam path as voltage change.

**Attachment of mosquitos to the flight mill:** Mosquitoes were immobilized by chilling in ice or anesthetized with CO_2_ for 1-2 minutes. As seen in Figure 2, a piece of 4 cm copper wire was attached to the dorsal thorax of individual mosquitos using UV-activated glue (Xfactor hydro sticky-Icky1000 LOCA UV glue, item model number SI2000g30, purchased from Amazon) and an UV flashlight. One end of the copper wire was inserted into the glass capillary lever arm of the flight mill. A small aluminum flag was attached to the other side of the capillary.

**Flight Protocol**: Mosquitoes that did not start to fly during 1 minute after attachment to the flight mill were discarded. After 1 minute on the flight mill, mosquitoes were monitored for the next five minutes. Mosquitoes that flew continuously during this time advanced to a 15 minutes test period during which flight data were collected. Temperature and humidity were monitored and ranged from 25-28 °C and 33-46 %, respectively.

**Automated data collection and analysis**: Collected data was then converted as a readout of flight duration, distance travelled, and speed. Raw data were initially transferred and analyzed in Microsoft Excel. Distance traveled during an individual flight test was calculated by multiplying the sum of revolutions per second by the circumference of the flight mill travel path (47 cm). MATLAB R2019a was used for graphing the data and to create flight models.


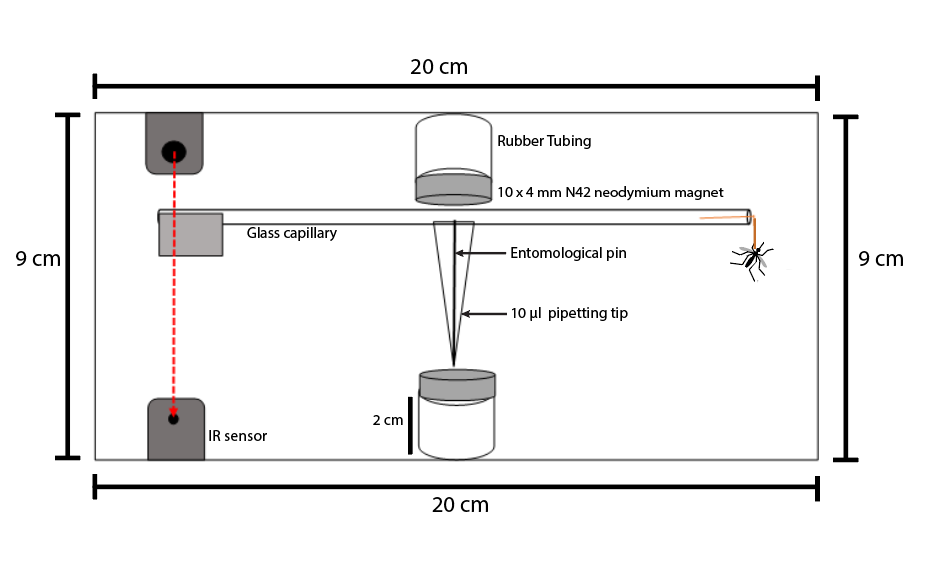


**Supplemental Figure 1.** **Schematic diagram of the flight mill –** A magnetic needle inside a pipette tip was held in between two magnets. The glass capillary attached to the pipette tip has aluminum foil attached to counter balance the mosquito that was attached to the other end. Passing of the aluminum foil attached at the end of a glass capillary breaks the beam path as the attached mosquito flies and rotates the arm. The IR-sensor was connected to an Arduino microcontroller to detect the break in the beam path as voltage change.


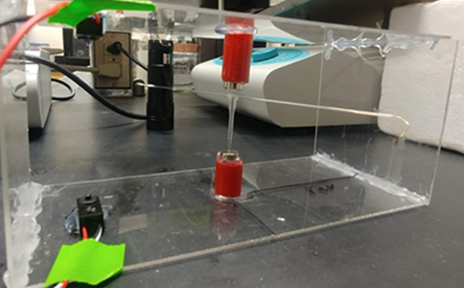


**Supplemental Figure 2. Construction of the flight mill** **–** The lever arm was attached to a 10 µl pipetting tip with an entomological metal pin placed inside to provide a pivot. This assembly was placed between two magnets held on the center of the floor and ceiling of acrylic sheets. The magnets were attached to rubber tubing. One end of the copper wire was inserted into the glass capillary lever arm of the flight mill. A small aluminum flag was attached to the other side of the capillary.


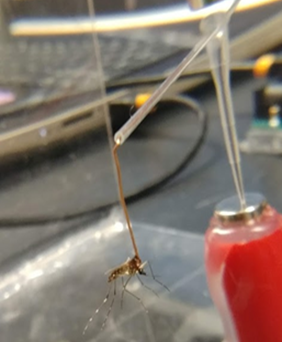


**Supplemental Figure 3. Attachment of mosquito to the flight mill –** A piece of 4 cm copper wire was attached to the dorsal thorax of individual mosquitos using UV-activated glue (ULV) and an UV flashlight. One end of the copper wire was inserted into the glass capillary lever arm of the flight mill.
